# Supplementary material for: Analysing Twitter semantic networks: the case of 2018 Italian elections
Source: Sci Rep. 2021 Jun 24;11:13207. doi: 10.1038/s41598-021-92337-2 (PMC8225802; doi:10.1038/s41598-021-92337-2)
Supplement: Supplementary file 1 — Supplementary Information. [file 41598_2021_92337_MOESM1_ESM.pdf]

Supplementary Information
Analysing Twitter Semantic Networks: the case of 2018 Italian Elections

Tommaso Radicioni[1,2,*], Fabio Saracco[2], Elena Pavan[3], and Tiziano Squartini[2]

[1]Scuola Normale Superiore, P.zza dei Cavalieri 7, 56126, Pisa, Italy
[2]IMT School for Advanced Studies, P.zza S. Ponziano 6, 55100, Lucca, Italy
[3]University of Trento, Via Verdi 26, 38122, Trento, Italy
[*]tommaso.radicioni@sns.it

Supplementary Note 1: Defining a similarity measure

A *sequence-based similarity* quantifies the cost of transforming a string x into a string y when the two strings are viewed as sequences of characters. String transformation is defined by three elementary operations: 1) deleting a character, 2) inserting a character and 3) substituting one character with another[1]. The edit distance function $d(x,y)$ aims at capturing the mistakes of human editing, such as inserting extra characters or swapping any two characters. To merge only strings that are either misspelled or different by number (i.e. singular in place of plural and viceversa) we have set the threshold for the maximum number of allowed differences between any two strings to 2.

Supplementary Note 2: Projecting and validating bipartite networks

As anticipated in the main text, the idea behind a filtered projection is that of *linking any two nodes belonging to the same layer if found to be sufficiently similar*. The steps to implement such a procedure are described below.

Quantifying nodes similarity. First, a measure quantifying the similarity between nodes is needed. Given any two nodes (say, α and β) we follow[2] and count the total number of common neighbors $V^*_{\alpha\beta}$, i.e.

$$V^*_{\alpha\beta} = \sum_{j=1}^{N_\top} m_{\alpha j} m_{\beta j} = \sum_{j=1}^{N_\top} V^j_{\alpha\beta} \tag{1}$$

the value of $V^j_{\alpha\beta}$ being 1 if nodes α and β share the node i as a common neighbor and 0 otherwise. Notice that the non-filtered projection of a bipartite network corresponds to a monopartite network (say, $\mathbf{A}$) whose generic entry reads $a_{\alpha\beta} = \Theta[V^*_{\alpha\beta}]$ (i.e. it is an edge in correspondence of any non-zero value of $V^*_{\alpha\beta}$).

Quantifying the statistical significance of nodes similarity. The statistical significance of any two nodes similarity is quantified with respect to a bunch of null models which will be now derived from first principles. To this aim, let us consider the maximization of Shannon entropy

$$S = -\sum_{\mathbf{G}\in\mathscr{G}} P(\mathbf{G}) \ln P(\mathbf{G}) \tag{2}$$

over the set of all, possible, bipartite graphs with, respectively, $N_\top$ nodes on one layer (say, users) and $N_\perp$ nodes on the other (say, hashtags). Since entropy-maximization will be carried out in a constrained framework, let us discuss each set of constraints separately.

Bipartite Configuration Model. The *Bipartite Configuration Model* (BiCM) represents the bipartite variant of the Configuration Model (CM). Upon introducing the Lagrangian multipliers $\boldsymbol{\theta}$ and $\boldsymbol{\eta}$ to enforce the proper constraints (i.e. the ensemble average of the degrees of users and hashtags, respectively $h^*_i = \sum_\alpha m_{i\alpha}$, $\forall\, i$ and $k^*_\alpha = \sum_i m_{i\alpha}$, $\forall\, \alpha$) and ψ to enforce the normalization of the probability, the recipe prescribes to maximize the function

$$\mathscr{L} = S - \psi\left[1 - \sum_{\mathbf{G}\in\mathscr{G}} P(\mathbf{G})\right] - \sum_{i=1}^{N_\top} \theta_i [h^*_i - \langle h_i\rangle] - \sum_{\alpha=1}^{N_\perp} \eta_\alpha [k^*_\alpha - \langle k_\alpha\rangle] \tag{3}$$

(with respect to $P(\mathbf{G})$. This leads to

$$P(\mathbf{G}|\boldsymbol{\theta},\boldsymbol{\eta})=\frac{e^{-H(\mathbf{G})}}{Z}=\prod_{i=1}^{N_\top}\prod_{\alpha=1}^{N_\perp}\left(\frac{x_i y_\alpha}{1+x_i y_\alpha}\right)^{m_{i\alpha}}\left(\frac{1}{1+x_i y_\alpha}\right)^{1-m_{i\alpha}}=\prod_{i=1}^{N_\top}\prod_{\alpha=1}^{N_\perp}p_{i\alpha}^{m_{i\alpha}}(1-p_{i\alpha})^{1-m_{i\alpha}} \tag{4}$$

where $x_i \equiv e^{-\theta_i}$ and $y_\alpha \equiv e^{-\eta_\alpha}$. The quantity $p_{i\alpha}=\frac{x_i y_\alpha}{1+x_i y_\alpha}$ can be interpreted as the probability that a link connecting nodes i and α is there; the matrix of probability coefficients $\{p_{i\alpha}\}$ induces the expected values $\langle h_i\rangle=\sum_\alpha p_{i\alpha}, \forall i$ and $\langle k_\alpha\rangle=\sum_i m_{i\alpha}, \forall\alpha$ and can be numerically determined by solving the set of $N_\top+N_\perp$ equations $\langle h_i\rangle=h_i^*, \forall i$ and $\langle k_\alpha\rangle=k_\alpha^*, \forall\alpha$.

According to the BiCM, the presence of each $V_{\alpha\beta}^j$ can be described as the outcome of a Bernoulli trial:

$$f_{\text{Ber}}(V_{\alpha\beta}^j=1) = p_{\alpha j}p_{\beta j}, \tag{5}$$

$$f_{\text{Ber}}(V_{\alpha\beta}^j=0) = 1-p_{\alpha j}p_{\beta j}. \tag{6}$$

The independence of links implies that each $V_{\alpha\beta}$ is the sum of independent Bernoulli trials, each one characterized by a different probability. The behavior of such a random variable is described by a Probability Mass Function (PMF) called Poisson-Binomial.

Bipartite Partial Configuration Model. The BiCM constrains the degrees of both the users and the hashtags. Such a model can be 'relaxed' by limiting ourselves to constrain the degrees of the nodes belonging to the layer of interest - in this case, the degrees of the hashtags. Upon 'switching off' the user-specific constraints, one end up with a simplified version of the BiCM, characterized by a generic probability coefficient reading $p_{i\alpha}=\frac{h_\alpha^*}{N_\top}$, in turn leading to the expression $f_{\text{Ber}}(V_{\alpha\beta}^j=1)=\frac{h_\alpha^* h_\beta^*}{N_\top^2}$. The evidence that the latter expression does not depend on j simplifies the description of the random variable $V_{\alpha\beta}$, now obeying a PMF called Binomial, i.e.

$$f_{\text{BiPCM}}(V_{\alpha\beta}=n)=\binom{N_\top}{n}\left(\frac{h_\alpha^* h_\beta^*}{N_\top^2}\right)^n\left(1-\frac{h_\alpha^* h_\beta^*}{N_\top^2}\right)^{N_\top-n}. \tag{7}$$

Bipartite Random Graph Model. The BiRG (Bipartite Random Graph) model is the bipartite variant of the traditional Random Graph Model. As for its monopartite counterpart, the probability that any two nodes are linked is equal for all the nodes and reads $p_{i\alpha}=\frac{N_\top N_\perp}{L}\equiv p_{\text{BiRG}}$ (where L is the empirical number of 'bipartite' edges). In this case, we have $f_{\text{Ber}}(V_{\alpha\beta}^j=1)=p_{\text{BiRG}}^2$ and the PMF describing the behavior of $V_{\alpha\beta}$ is a Binomial, i.e.

$$f_{\text{BiRG}}(V_{\alpha\beta}=n)=\binom{N_\top}{n}(p_{\text{BiRG}}^2)^n(1-p_{\text{BiRG}}^2)^{N_\top-n}. \tag{8}$$

Validating the monopartite projection. The statistical significance of the similarity of nodes α and β, thus, amounts at computing a p-value on one of the aforementioned probability distributions, i.e. the probability of observing a number of V-motifs greater than, or equal to, the observed one:

$$\text{p-value}(V_{\alpha\beta}^*)=\sum_{V_{\alpha\beta}\geq V_{\alpha\beta}^*} f(V_{\alpha\beta}). \tag{9}$$

After this procedure is repeated for each pair of nodes, an $N_\perp\times N_\perp$ matrix of p-values is obtained. The choice of which p-values to retain has to undergo a validation procedure for testing multiple hypotheses at the same time: here, the False Discovery Rate (FDR) procedure is used. The m p-values (in our case, $m=N_\perp(N_\perp-1)/2$) are, first, sorted in increasing order, $\text{p-value}_1\leq\ldots\leq\text{p-value}_m$ and, then, the largest integer $\hat{i}$ satisfying the condition

$$\text{p-value}_{\hat{i}}\leq\frac{\hat{i}t}{m} \tag{10}$$

(where t represents the single-test significance level - in our case, set to 0.05) is individuated. All p-values that are less than, or equal to, $\text{p-value}_{\hat{i}}$ are kept, i.e. all node pairs corresponding to those p-values will be linked in the resulting monopartite projection.

Supplementary Note 3: Analysing a network mesoscale structure

Community detection: the Louvain algorithm

After the daily monopartite user networks have been obtained, the Louvain algorithm[3] has been run to detect the presence of communities. This algorithm works by searching for the partition attaining the maximum value of the modularity function Q, i.e.

$$Q = \frac{1}{2L}\sum_{i,j}\left[a_{ij} - \frac{k_i k_j}{2L}\right]\delta_{c_i,c_j} \tag{11}$$

a score function measuring the optimality of a given partition by comparing the empirical pattern of interconnections with the one predicted by a properly-defined benchmark model. In the expression above, a_{ij} is the generic entry of the network adjacency matrix $\mathbf{A}$, the factor $\frac{k_i k_j}{2L}$ is the probability that nodes i and j establish a connection according to the Chung-Lu model, $\boldsymbol{c}$ is the N-dimensional vector encoding the information carried by a given partition (the i-th component, c_i, denotes the module to which node i is assigned) and the Kronecker delta δ_{c_i,c_j} ensures that only the nodes within the same modules provide a positive contribution to the sum. The normalization factor $2L$ guarantees that $-\frac{1}{4} \leq Q(\boldsymbol{c}) \leq 1$. Moreover, a reshuffling procedure has been applied to overcome the dependence of the original algorithm on the order of the nodes taken as input.

Core-periphery detection

Core-periphery detection can be carried out upon adopting the method proposed in[4] and prescribing to search for the network partition minimizing the quantity called *bimodular surprise*, i.e.

$$\mathscr{S}_{\|} = \sum_{i \geq l^*_\bullet}\sum_{j \geq l^*_\circ}\frac{\binom{V_\bullet}{i}\binom{V_\circ}{j}\binom{V-(V_\bullet+V_\circ)}{L-(i+j)}}{\binom{V}{L}}; \tag{12}$$

as anticipated in the main text, L is the total number of links, while V is the total number of possible links, i.e. $V = \frac{N(N-1)}{2}$. The quantities marked with $\bullet$ ($\circ$) refer to the corresponding core (periphery) quantities, i.e. $V_\bullet$ is the total number of possible core links, $V_\circ$ is the total number of possible periphery links, $l^*_\bullet$ is the number of observed links within the core and $l^*_\circ$ is the number of observed links within the periphery.

From a technical point of view, $\mathscr{S}_{\|}$ is the p-value of a multivariate hypergeometric distribution, describing the probability of $i+j$ successes in L draws (without replacement), from a finite population of size V that contains exactly $V_\bullet$ objects with a first specific feature and $V_\circ$ objects with a second specific feature, wherein each draw is either a 'success' or a 'failure': analogously to the univariate case, $i+j \in [l^*_\bullet + l^*_\circ, \min\{L, V_\bullet + V_\circ\}]$. The method outputs the most statistically significant core-periphery structure compatible with the network under analysis.

Supplementary Note 4: Computing the polarization of non-verified users

Let C_c, with $c = 1,2,3$, indicate the set of (both verified and non-verified) users belonging to community c and N_α, with $\alpha = 1,2,3$ the set of neighbours of verified users belonging to the community $c = \alpha$. A non-verified user *polarization* is defined as

$$\rho_\alpha = \max_c\{I_{\alpha c}\} \tag{13}$$

where

$$I_{\alpha c} = \frac{|C_c \cap N_\alpha|}{|N_\alpha|}. \tag{14}$$

As it has been shown in[5], the polarization index reveals how unbalanced is the distribution of interactions between non-verified users and verified users: non-verified accounts basically focus their retweeting activity on the tweets of verified users within the same community, thus providing a clear indication of the community of which a non-verified user is likely to be a member.

References

1. Levenshteĭn, V. I. Binary codes capable of correcting deletions, insertions, and reversals. Sov. Phys., Dokl. 10 (1965), 707-710 (1966); translation from Dokl. Akad. Nauk SSSR 163, 845-848 (1965). (1965).
2. Saracco, F. *et al.* Inferring monopartite projections of bipartite networks: an entropy-based approach. *New J. Phys.* **19**, 053022, DOI: 10.1088/1367-2630/aa6b38 (2017).
3. Blondel, V. D., Guillaume, J., Lambiotte, R. & Lefebvre, E. Fast unfolding of communities in large networks. *J. Stat. Mech. Theory Exp.* **2008**, P10008, DOI: 10.1088/1742-5468/2008/10/p10008 (2008).
4. de Jeude, J. v. L., Caldarelli, G. & Squartini, T. Detecting core-periphery structures by surprise. *Europhys. Lett.* **125**, 68001 (2019).
5. Becatti, C., Caldarelli, G., Lambiotte, R. & Saracco, F. Extracting significant signal of news consumption from social networks: the case of Twitter in Italian political elections. *Palgrave Commun.* DOI: 10.1057/s41599-019-0300-3 (2019). 1901.07933.
